# miR‐495 sensitizes MDR cancer cells to the combination of doxorubicin and taxol by inhibiting MDR1 expression

**DOI:** 10.1111/jcmm.13114

**Published:** 2017-04-14

**Authors:** Zhenyou Zou, Ruyi Zou, Dan Zong, Yonghong Shi, Jinyao Chen, Jie Huang, Jiahui Zhu, Liguan Chen, Xiaoyan Bao, Yuan Liu, Weihao Liu, Wenhui Huang, Jingsang Hu, Zhi Chen, Xiaojie Lao, Chaoqun Chen, Xiaoli Huang, Yao Lu, Xueyin Ni, Daoquan Fang, Dengqiang Wu, Shuangshuang Lu, Mingzhu Jiang, Chenyang Qiu, Yuya Wu, Qisha Qiu, Yanyuan Dong, Yangyang Su, Chenmin Zhao, Zhihe Zhong, Jing Cai, Yong Liang

**Affiliations:** ^1^ Tumor Institute Taizhou University Taizhou ZJ China; ^2^ Biochemistry Department of Purdue University West Lafayette IN USA; ^3^ Chemistry Department of Shangrao Normal University Shangrao JX China; ^4^ Life science College of Nanjing Agricultural University Nanjing JS China; ^5^ Radiology Department of Taizhou Hospital Taizhou ZJ China

**Keywords:** miR‐495, multidrug resistance, multidrug resistance protein 1

## Abstract

MDR1 is highly expressed in MDR A2780DX5 ovarian cancer cells, MDR SGC7901R gastric cancer cells and recurrent tumours. It pumps cytoplasmic agents out of cells, leading to decreased drug accumulation in cells and making cancer cells susceptible to multidrug resistance. Here, we identified that miR‐495 was predicted to target *ABCB1*, which encodes protein MDR1. To reduce the drug efflux and reverse MDR in cancer cells, we overexpressed a miR‐495 mimic in SGC7901R and A2780DX cells and in transplanted MDR ovarian tumours *in vivo*. The results indicated that the expression of MDR1 in the above cells or tumours was suppressed and that subsequently the drug accumulation in the MDR cells was decreased, cell death was increased, and tumour growth was inhibited after treatment with taxol‐doxorubicin, demonstrating increased drug sensitivity. This study suggests that pre‐treatment with miR‐495 before chemotherapy could improve the curative effect on MDR1‐based MDR cancer.

## Introduction

Cancer is responsible for millions of deaths each year 1. Chemotherapy is the most commonly adopted method to treat this devastating disease, but the development of multidrug resistance (MDR) can protect malignant cells from drugs, thereby greatly weakening the efficiency of the treatment 2.

Enzymes, anti‐apoptotic genes and DNA repair mechanisms reportedly contribute to MDR 3, 4. P‐gp, also named MDR protein 1 (MDR1, a membrane‐associated protein encoded by the *ABCB1* gene), can remove intracellular drugs from cells. Therefore, the overexpression of MDR1 decreases drug accumulation and makes cells susceptible to MDR. For these reasons, several therapies have been focused on the inhibition of *ABCB1*. To date, three generations of MDR1 inhibitors have been developed since 1981, when Tsuruo *et al*. found that the MDR of vincristine‐resistant P388/VCR leukaemia cells could be reversed by verapamil 5. However, none of these treatments have been clinically successful because of their unwanted pharmacokinetic interactions with chemotherapeutic agents or because of a lack of specificity 6.

MicroRNAs are a type of small, non‐coding, single‐stranded RNA. Through complementary binding to the 3′ untranslated region (3′‐UTR) of their respective target mRNAs, microRNAs are able to silence genes and play critical roles in the regulation of cell functions 7. MDR1 is reported to be regulated by different miRNAs in different tumour types. For example, miR‐451 negatively regulates the expression of the MDR1 gene in the multidrug‐resistant breast cancer cell line MCF7/DOX 8. The growing evidence demonstrating the regulation of MDR1 expression by miRNAs led us to investigate the possibility of using a miR‐based approach to silence MDR1 overexpression in human multidrug‐resistant cancer cells.

miR‐495 possesses 22 alkali bases. By interacting with mRNAs, miR‐495 affects cells in various aspects. Here, through the combined use of the online computational algorithms TargetScan, PicTar, DIANA‐microT, miRDB, miRWalk‐validated, miR2Disease and Target mRNA, we identified that miR‐495 is predicted to target *ABCB1* (Figure 2). Therefore, the reduced expression of MDR1 *via* the complementary binding of miR‐495 to the mRNA of MDR1 could decrease drug efflux from the cell, improve the chemotherapeutic effect and reverse MDR in cancer.

In the following study, we selected two MDR cell lines, A2780DX5 and SGC7901R, that originated from human ovarian and gastric cancer, respectively, and that are resistant to doxorubicin and taxol because of their high expression of MDR1 7. We first transfected excess amounts of a synthesized mature miR‐495 mimic into the cells and then assayed the changes in MDR1 expression, drug accumulation and apoptosis following treatment with the combination of taxol‐doxorubicin chemotherapy. Finally, using xeno‐MDR tumour‐implanted mice, we observed slowed tumour growth induced by the anticancer drug combination therapy after miR‐495 administration.

## Materials and methods

### Materials

Paclitaxel (Taxol, CAS: 33069‐62‐4), doxorubicin (CAS: D1515) and cisplatin (CAS: D15663‐27‐1) were purchased from Sigma‐Aldrich. FITC‐labelled paclitaxel, which was used as an indicator of cytoplasmic drug accumulation, was donated by Dr. Han Zou of Nanjing University. The synthetic mature miR‐495 mimic (CAS: hsa‐miR‐495) and the nonsense RNA were purchased from Cell Biolabs Inc. (San Diego, CA, USA). The antibodies against MDR1 (CAS: sc‐13131) and GAPDH (CAS: sc‐32233) were obtained from Santa Cruz Biotech (Santa Cruz, CA, USA). The plasmids pSi‐ABCB1siRNA, which targets ABCB1, and pSi‐miR‐495 sensor, along with their respective negative control pSi‐negatives, were provided by Genepharm (Pallini, Greece). The p‐MIR‐reporter plasmid and β‐galactosidase (β‐gal) expression plasmid were bought from Ambion (Grand Island, NY, USA). Luciferase Reporter Assay Kits were purchased from BioVision Inc. (Cat: K801‐200; Milpitas, CA, USA) and Promega (Cat: E1483; Madison, WI, USA). In addition, five primary ovarian and six primary gastric cancer samples were obtained from the excised tissue tumour tissues donated by cured patients in Taizhou municipal hospital, and recurrent ovarian and gastric tumour tissues were independently obtained from five patients with ovarian cancer and six patients with gastric cancer.

### Cell selection for the MDR study

To obtain the appropriate cancer cells for the MDR study, four cell lines were used. Two of the cell lines, the ovarian‐originated A2780 cancer cells (KeyGen Biotech, Nanjing, China) and the gastric‐originated SGC7091 cancer cells (American Type Culture Collection, Manassas, VA, USA), expressed low levels of MDR1, while the other two cell lines, the MDR ovarian cancer cell line A2780DX5 (KeyGen Biotech) and MDR gastric cancer cell line SGC7901R (American Type Culture Collection) expressed high levels of MDR1. In the low MDR1‐expressing cells, we examined the increase in drug resistance after MDR1 enhancement. In addition, in the MDR1‐rich cells, we investigated the decrease in drug resistance when MDR1 was inhibited. In addition, the cisplatin‐resistant (cisplatin is not a substrate of MDR1) but taxol‐sensitive cell line A2780C (American Type Culture Collection) was used to ensure that miR‐495 is ABCB1 specific and that the effect of the non‐MDR1 substrate drugs is not also enhanced by the miR‐495 overexpression.

Three weeks before the experiment, all of the tested cell lines were cultured in RPMI 1640 medium with 10% foetal bovine serum (FBS) and 1% penicillin/streptomycin at 37°C in a humidified atmosphere containing 5% CO_2_. For the MDR cell lines A2780DX5, SGC7901R and A2780C, a low dose of doxorubicin and taxol (2.5 μg/ml doxorubicin and 0.05 μg/ml taxol) or cisplatin (0.05 μg/ml) was added to the culture medium to maintain the MDR characteristics of the cells.

The above cell lines (except A2780C) were then subcultured in drug‐free complete medium for 12 hrs, after which different, gradually increasing concentrations of drug mixtures (from 0 to 40 μg/ml, with a ratio of doxorubicin to taxol of 20:1) were added to the media in different culture dishes. Fifteen hours (hrs) later, the cells were harvested. The levels of MDR1 and GAPDH in both the drug‐treated cells and five primordial or five recurrent ovarian and gastric cancer samples from patients were independently analysed by Western blotting. The levels of miR‐495, MDR1 and GAPDH mRNA were measured by RT‐qPCR. Drug‐induced apoptosis was determined by flow cytometry and caspase activity analysis, and the cell viability and proliferation were measured using an MTT kit at 5, 10, 15, 24, 48 hrs or by 0.4% trypan blue staining at 15 hrs.

The other MDR cell line, A2780DX5, and the corresponding sensitive cell line A2780 were additionally cultured for the drug accumulation assay. In this assay, FITC‐labelled taxol was used as the indicator drug. It was administered in the medium at the typical taxol dose of 2 μg/ml, and the bright spots observed under a confocal microscope (LEXT OLS41003D; Olympus, Tokyo, Japan) were the sites where FITC‐taxol was located. The fluorescence intensity was measured by a FACScan flow cytometer (BD FACSCanto II; Becton‐Dickinson, Franklin Lakes, NJ, USA) and used to quantify the concentration of FITC‐taxol taken up by the cells.

### Prediction of microRNAs targeting ABCB1

The online algorithms ‘PicTar’ (http://www.pictar.org/), ‘TargetScan Human 6.2′ (http://www.targetscan.org/), ‘Target mRNA’ (http://www.microrna.org/), DIANA‐microT, miRDB, miRWalk‐validated and miR2Disease were combined to search the microRNAs targeting the MDR1 gene (ABCB1). The mirSVR and PhastCons scores were used to value the hybrid affinities. Pearson's correlation scatter plots were employed to judge the correlation between the levels of the predicted microRNAs and MDR1 protein/mRNA after analysing the dose‐dependent effects of a miR‐495 mimic on MDR1 protein and mRNA expression by Western blotting and RT‐qPCR, respectively.

### Luciferase reporter assay

To confirm the target prediction and to test the direct binding of microRNA, the entire 3′‐UTR of human ABCB1 was PCR‐amplified (using primer FW: 5′‐TGAGACTGACATACTCTACA‐3′ and primer RW: 5′‐GTTCTTAGTCGTCCTAG‐3′), PmeI and HindIII restriction enzyme sites were introduced to either end of the product with their corresponding adapters, and the PCR product was then inserted into the MCS of the p‐MIR‐reporter plasmid.

A luciferase reporter plasmid with a mutated ABCB1 3′UTR sequence insert was used to determine the specificity of the binding between miR‐495 and the ABCB1 3′‐UTR. The original sequence that interacted with the microRNA seed sequence was mutated from UUUGUU to AAACAA.

A2780DX5 cells, which exhibit high MDR1 expression, were divided into five groups and transfected with a firefly luciferase reporter plasmid, β‐galactosidase (β‐gal) expression plasmid, and equal amounts of either pre‐miR‐495, anti‐miR‐495 (the sequence is 5′‐AAGAAGUGCACCAUGUUUGUUU‐ 3′) or the scrambled negative control RNA (the sequence is 5′‐GACCUUCAUGUACCUGGCACCG‐3′). Twenty‐four hours later, the cells were assayed using a luciferase assay kit. The luciferase activity was reported in relative light units, and the firefly luciferase activity was normalized against the Renilla luciferase activity. All transfections experiments were performed in triplicate and repeated at least thrice in independent experiments.

### Analysis of the reversal of MDR in cancer cells by miR‐495

Before the formal experiment, the dose‐dependent effect of the miR‐495 mimic on MDR1 expression and the half‐life of the mimic was analysed using Western blotting and RT‐qPCR, respectively, and the miR‐495 mimic concentration that was able to inhibit MDR1 was then used in the following experiments.

To verify the effect of miR‐495 on MDR1, the taxol‐doxorubicin‐resistant cell lines A2780DX5 and SGC7901R were cultured in subgroups. The first cell group was cultured with complete medium and was set as the control; the second was transfected with 100 ng/ml of the miR‐495 mimic (which is sufficient to inhibit MDR1); the third was treated with a mixture of 10 μg/ml of doxorubicin and 1.25 μg/ml of taxol; and the fourth was cultured with the same doses of taxol and doxorubicin but had a 5‐hrs pre‐transfection with the miR‐495 mimic.

As controls, antisense (the sequence is 5′‐GUACUACUUGAAGCUAGCUGGC‐3′) and nonsense RNA mimics (the sequence is 5′‐GUACUACUUGAAGCUAGCUGGC‐3′) were administered to normally cultured or drug‐treated cells.

The non‐MDR1 substrate drug cisplatin was also administered to cells with and without pre‐transfection of miR‐495 to confirm that the chemotherapeutic effect is MDR1 specific.

For counterevidence, rifampicin (an MDR1 inducer that triggers ABCB1 expression *via* the pregnane X receptor‐mediated signalling pathway 9, at a dose of 20 μg/ml) was administered to A2780 cells, which express a low level of MDR1 and are thereby sensitive to taxol/doxorubicin, 2 weeks before the experiment to verify that the drug efflux caused by an increase in MDR1 expression can protect cells from drug‐induced cell death and that can confer a drug‐resistant phenotype to the sensitive cells.

After 15 hrs of culture, all of the cells were harvested and subjected to various assays.

### RNAi

To test the hypothesis that the effects of MDR reversal are caused by the inhibition of MDR1 and to demonstrate that the drug efflux can be reduced, thus improving the anticancer effect of chemotherapeutic drugs, siRNA duplexes were designed to target the coding region of the MDR1 mRNA at nt 1545–1565 with the sequences 5′‐GUAUUGACAGCUAUUCGAAdTdT‐3′ and 3′‐dTdTCAUAACUGUCGAUAAGCUU‐5′. DNA oligonucleotides were synthesized and inserted into a BamHI‐HindIII linearized pSilencer 2.1‐U6 hygro vector according to the manufacturer's instructions. The sequence of the resulting hairpin siRNA is 5′‐GTATTGACAGCTATTCGAAGAGTGGGCAttcaagagaTGCCCACTCTTCGAATAGCTGTCAATACttt‐ttt‐3′. A phosphorothioate antisense oligonucleotide (the sequence was 5′‐CCATCCCGACCTCGCGCTCC‐3′) bracketing the start codon of the MDR1 transcript was inserted into the corresponding sites of the vector and served as the negative control plasmid.

The MDR1 siRNA expression plasmid (100 ng/ml, Genepharm Inc.) and the negative control plasmid (Genepharm Inc.) were independently cotransfected with the same quantities of Renilla luciferase reporter vector, pRL‐TK and pGL3‐GFPf into A2780DX5 cells (6 × 10^5^ cells in 200 μl of culture medium). Forty‐eight hours later, the cells were lysed and analysed for their levels of firefly and Renilla luciferase activity.

Parallel cells were subjected to protein and mRNA extractions for the detection of MDR1 protein and MDR1 mRNA expression, respectively. A subgroup of cells was cultured for an additional 15 hrs in taxol‐doxorubicin‐containing medium. Then, the apoptosis and drug uptake of the MDR1 RNAi cells were analysed as described below.

### Apoptosis assay

Cell apoptosis was determined by a FACScan flow cytometer using an Annexin‐V‐FITC apoptosis detection kit (Becton‐Dickinson) according to the manual. The results were calculated using the CellQuestTM Pro software (Becton‐Dickinson), and Annexin‐V positive dots were counted as apoptotic cells.

### Caspase‐3 activity assay

The activity of caspase‐3 was determined using the corresponding caspase activity detection kits (R&D Systems, Minneapolis, MN, USA). Briefly, 100 μg of total protein was added to 50 μl of reaction buffer, and 5 μl of the substrates DEVD‐pNA and LEDHpNA were used to analyse the activity of caspase‐3. The samples were incubated at 37°C for 3 hrs, and the enzyme‐catalysed release of pNA was quantified at 405 nm using a microtitre plate reader. The values of the treated samples were normalized to the corresponding untreated controls to determine the fold increase or decrease in caspase activity.

### MTT assay

Cells were plated at 10^5^ per well in a 96‐well plate and treated with the taxol‐doxorubicin mixture for 5, 10, 15, 24 and 48 hrs, with a drug blank group as the control. Ten microlitres of the MTT reagent (ATCC, CAT: ATCC 30‐1010K) was added to each well and incubated for 4 hrs. Then, 100 μl of the detergent reagent was added and incubated at room temperature in the dark for 2 hrs. Finally, the absorbance of each well was assayed using a microplate reader (SpectraMax M5; Molecular Devices Corp., Boston, MA, USA) at 570 nm.

### Trypan blue exclusion assay

Cell viability was determined by trypan blue (0.04%) staining and direct cell counting using a hemacytometer after drug, miR‐495 or siRNA administration. The cells lacking trypan blue dye were defined as viable cells.

### Animal experiments

In strict accordance with the Guidance for the Care and Use of Laboratory Animals of the National Institutes of Health and the protocol approved by the Committee on the Ethics of Animal Experiments of Taizhou University (Permit Number: 13‐1368), BALB/c mice were subaxillary hypo‐injected with A2780DX5 MDR cells (200 μl for each mouse at a titre of 10^7^/ml).

When the tumours reached 6 mm in diameter, vein injections of the miR‐495 mimic along with Lipofectamine 2000 were performed, and the half‐life of the miR‐495 mimic in the serum of the mice was assayed. Specifically, the mRNA was extracted from the serum, and the concentration of miR‐495 in the serum was measured at 2, 6, 12, 24, 48, 72 and 96 hrs after injection by RT‐QPCR. When the miR‐495 in the serum was half of that observed at 2 hrs, this duration was chosen as the interval between two administrations. The dose of the miR‐495 mimic used in the animals was also determined by testing increasing concentrations of the miR‐495 mimic. When the drug caused the expression of the MDR1 protein to disappear, as analysed by Western blot, this quantity was used to treat the animals. After that, the tumour‐bearing mice were separated into six groups of five mice each. In the first group, each mouse was tail‐vein injected with 200 μl of physical saline every other day. Based on our previous experiments and reference 10, the mice in the second group were injected with 200 μl of the drug mixture (590 μg/ml of doxorubicin and 10 μg/ml of taxol) with an interval of 1 day between each treatment. In the third group, each mouse was hypodermically injected with 200 μl of PEI (polyetherimide)‐conjugated miR‐495 mature sequence mimic near the tumours every other day (2 μg/ml, the dosage and the administration intervals were determined according to the MDR1 suppression and half‐life assays). The fourth group was injected with nonsense RNA. The fifth group received injections of either the drug mixture or the miR‐495 mimic on alternate days. The sixth group received injections of either the drug mixture or nonsense RNA on alternate days.

To verify that MDR1 is regulated by miR‐495 *via* complementary binding to the 3′‐UTR, plasmids containing the MDR1 complementary DNA (cDNA) but lacking the 3′‐UTR were constructed and administered to the tumours treated or untreated with the drug mixture using Lipofectamine 2000. Specifically, the MDR1 cDNA was cloned into the pGEM3Zf (‐) vector using EcoR1 digestion. The 3′‐UTR‐free MDR1 cDNA fragment was excised by digestion with Xba1 and blunt‐ended using the DNA Pol I Klenow fragment (Pharmacia, Buckinghamshire, UK) according to the manufacturer's instructions. The fragment was cloned into the HpaI site of the pRUFneo retroviral expression vector21 and checked for the correct orientation. The pRUFneo (MDR1 lacking 3′‐UTR) construct was expanded in DH10 *Escherichia coli* cells, and the plasmids were purified using a midi‐prep kit (Qiagen‐12143, Hilden, Germany).

The above assays and tumour size measurements were performed every other day. After 10 days, all of the mice were euthanized under sodium pentobarbital anaesthesia. The tumours were removed. After volume determination, sections of the tumours were sliced and stained *in situ* for miR‐495. MDR1 and miR‐495 were also quantitatively measured by Western blotting and RT‐qPCR.

### microRNA mimic transfection

The transfection of miRNA mimics was performed using the HiPerFect transfection reagent according to the manufacturer's protocol. Briefly, RNA mimics (100 ng/ml) diluted in DEPC–water were mixed with Lipofectamine 2000 (40 μl/ml) and incubated at room temperature for 20 min. Then, 100 ml/well of the mixture was added to cells in FBS‐free medium. After mixing, the cell plates were kept in an incubator (WJ‐2‐80; Hengyu Inc., Shanghai, China) for cell culture as previously described.

### Western blotting

The tumour tissues were ground on ice for 30 min., and then along with the cultured cells, they were lysed in RIPA (Radio‐Immunoprecipitation Assay) buffer containing protease inhibitors and PMSF (Cat: P0013C; Biotime Inc, Nantong, China) at 4°C for 30 min. Equal amounts (60 μg) of cell lysates were resolved by SDS‐PAGE and transferred to PVDF membranes (Amersham Biosciences). The PVDF membranes were incubated in TBS (Cat: T1085; Biomart Inc, Beijing China) for 1 hr and then blotted overnight at 4°C with an antibody against MDR1. The bound antibodies were detected by enzyme‐linked chemiluminescence (ECL; Pierce Biotechnology Inc., Rockford, IL, USA). Equal protein loading was monitored by probing the same samples with an antibody against GAPDH.

### Real‐time quantitative PCR

Total RNA from the above samples was independently extracted using TRIzol according to the product manual. After concentration determination, real‐time qPCR was performed in triplicate using a TaqMan PCR kit (TaqMan: CAS: N8080228) on an Applied Biosystems 7500 Sequence Detection System (Applied Biosystems, Foster City, CA, USA) under the following conditions: 10 min. at 95°C, followed by 42 temperature cycles of 15 sec. at 95°C and 1 min. at 60°C. The primers used in the experiments were as follows: MDR1 primer FW: 5′‐CCCATCATTGCAATAGCAGC‐3′; RW: 5′‐CTTCAAACTTCTGCTCCTGA‐3′. GAPDH primer FW: 5′‐TGCACCACCAACTGCTTAGC‐3′; RW: 5′‐GGCATGGACTGTGGTCATGAG‐3′. miR‐495 primer FW: 5′‐GCGGAAACAAACATGGTGCA‐3′; and miR‐49‐ primer RW:5′‐GTTGGCTCTGGTGCAGGGTCCGAGGTATTCGCACCAGAGCCAACAAGAAG‐3′.

### MiR‐495 *in situ* staining

To confirm that miR‐495 was transfected into the tumours, *in situ* hybridization and staining were performed. Briefly, frozen tumour sections placed on slides were pre‐treated with 0.2 M HCl, washed in 2 mg/ml glycine‐PBS and acetylated in a 0.1 M triethanolamine (pH 8.0) 0.25% acetic anhydride solution. The slides were heated to 90°C for 5 min., cooled over ice and pre‐hybridized in a solution containing 50% formamide, 0.6 M NaCl, 2 mM Tris‐HCl, pH 7.4, 1 mM EDTA, 1.0 mg/ml BSA, 0.02% PVP, 0.02% Ficoll, 10 mM DTT, 0.2 mg/ml ssDNA and 10% dextran sulphate. The hybridization was performed at 45°C overnight in a mixture containing a pre‐hybridization solution and 300 ng of digoxigenin‐labelled sense or nonsense miR‐495 probes (the sense and nonsense sequences are 5′‐AAGAAGUGCACCAUGUUUGUUU‐3′ and 5′‐GACCUUCAUGUACCUGGCACCG‐3′, respectively, Dako, Santa Barbara, CA, USA) for each sample. After hybridization, the samples were washed in 4 × SSC, and the unbound RNA probe was removed with RNase treatment. The samples were washed at a final stringency of 0.1 × SSC, 1 mM EDTA, and 1 mM DTT at 45°C. The DIG‐labelled miR‐495 probe was detected with a sheep antidigoxigenin antibody (dilution 1:100) coupled to alkaline phosphatase (Roche Diagnostics, Basel, Switzerland), which was used with a colour‐forming substrate solution according to the protocol provided by the manufacturer.

### Statistical analysis

All of the data were presented as the means ± S.E.M.s of three or more independent experiments. The statistical significance was calculated with Student's *t*‐test (paired, one tailed), and the differences were considered statistically significant at *P* < 0.05.

## Results

### MDR cells can tolerate a high dosage of combination drug treatment and express high levels of MDR1

**Figure 1:**
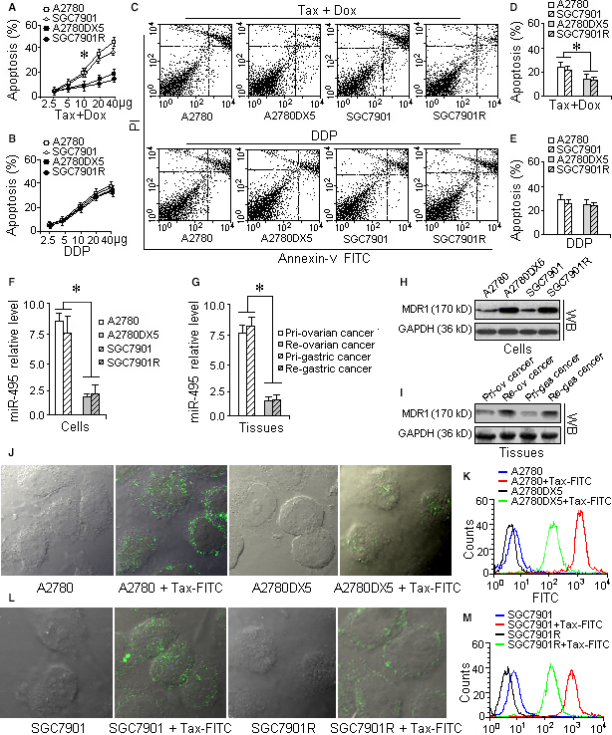
A2780DX5 and SGC7901R cells are more resistant to the combination of taxol and doxorubicin and express more MDR1 than the sensitive cell lines A2780 and SGC7901. To determine which cells were more resistant to the drug mixture, four cell lines were treated with gradually increasing doses. The cell line with the least apoptosis, as assayed by flow cytometry, could survive at a higher dose of the drug mixture and can be regarded resistant cells. If the MDR was caused by the expression of MDR1, the MDR cells might express more MDR1, so we assayed the protein and mRNA levels of MDR1 by Western blot and rt‐QPCR analysis, respectively. As an indicator for drug accumulation, we treated the cells with FITC‐labelled taxol to demonstrate that the cells with the highest expression of MDR1 also pumped out more of the chemotherapeutic drug, resulting in lower drug accumulation and causing MDR. (**A**) Dose‐dependent apoptosis of the MDR cell lines A2780DX5 and SGC7901R and drug‐sensitive cell lines A2780 and SGC7901 treated with the taxol‐doxorubicin combination. A2780DX5 and SGC7901R cells can resist higher concentrations of the drug combination than the A2780 and SGC7901 cells. (**B**) Dose‐dependent apoptosis of the MDR cell lines A2780DX5 and SGC7901R and the sensitive cell lines A2780 and SGC7901 treated with cisplatin. Cisplatin is not the substrate of MDR1; hence, the apoptosis of the four cell lines did not significantly differ. (**C**) Treatment with a 10 μg/ml dose of the taxol‐doxorubicin mixture for 15 hrs resulted in approximately 22% apoptosis in the sensitive cell lines A2780 and SGC7901 but only caused a 13% cell death rate in the MDR cell lines A2780DX5 and SGC7901R. However, 20 μg/ml of cisplatin only resulted in a 28% apoptosis rate, with no significant differences among the four tested cell lines. (**D**) The quantification of the apoptosis caused by the taxol‐doxorubicin combination in the four tested cell lines. The A2780DX5 and SGC7901R cells could resist a higher dose of the drugs than the A2780 and SGC7901 cells. (**E**) The quantification of the apoptosis caused by cisplatin alone in the four tested cells lines, A2780, SGC7901, A2780DX5 and SGC7901R. (**F**) The MDR cell lines A2780DX5 and SGC7901 expressed less miR‐495 than the sensitive cell lines. (**G**) Recurrent ovarian and gastric tumours expressed less miR‐495 than the primordial tumours of cancer patients. (**H**) The MDR1 levels in the MDR cells A2780DX5 and SGC7901R are higher than those in the sensitive cells A2780DX5 and SGC7901. (**I**) The MDR1 levels in the recurrent ovarian and gastric tumours are higher than those in the corresponding primordial tumours. (**J** and **L**) The FITC‐labelled anticancer drug taxol (bright green spots) accumulated less in the A2780DX5 and SGC7901R cells. (**K** and **M**) The drug fluorescence (the green histogram) is weaker compared with the drug‐sensitive A2780 cells (the red curve), indicating that more FITC‐taxol may have been pumped out of the cells. *Student's *t‐*test *(paired, one tailed), P <* 0.05*, n =* 5. The following conditions are the same.

Of the tested cell lines, A2780DX5 and SGC7901R cells are more tolerant of the taxol and doxorubicin mixtures than A2780 and SGC7901. As shown in Figure 1A–E, when the concentration of the taxol‐doxorubicin mixture in the medium reached 10 μg/ml, more than 22% of the A2780 and SGC7901 cells died in 15 hrs. In addition, the activity of caspase‐3 in the A2780 and SGC7901 cells was nearly 40% higher than in A2780DX5 and SGC7901R cells stressed with the drug mixture for 5 hrs, as shown in Figure S1A. In contrast, the A2780DX5 and SGC7901R cells had a 13% lower apoptosis rate with the same dose, and the caspase‐3 activity was lower than the A2780DX5 and SGC7901R cells (Fig. S1A). When the dose reached 40 μg/ml, nearly 40% of the A2780 and SGC7901 cells died, whereas only 19% of the A2780DX5 and SGC7901R cells died (Fig. 1A–E). An MTT assay further confirmed that the A2780DX5 and SGC7901R cells are more resistant than the A2780 and SGC7901 cells because their viability and proliferation decreased more slowly than that of the A2780 and SGC7901 cells in response to the increased drug doses (Fig. S1B).

With regard to cisplatin, significant differences in resistance among the above four cell lines were not observed. In the 15 hrs of cisplatin treatment, more than 28% of the above cells had died, and the caspase‐3 activities were all in the range of 0.7–0.9 OD (Fig. S1A). An MTT assay also demonstrated that the therapeutic effect of the non‐MDR1‐substrate drug cisplatin was not affected by overexpression of the miR‐495 mimic, suggesting that miR‐495 is MDR1 specific (Fig. S1C).

An RT‐PCR assay indicated that the levels of miR‐495 were lower in the A2780DX5 and SGC7901R cells and that the recurrent tumours of cancer patients behaved comparably to the A2780 and SGC7901 cells and the primordial ovarian or gastric cancer samples (Fig. 1F and G). Western blot analysis showed that the resistant cells and the recurrent tumours had higher levels of MDR1 expression than the sensitive A2780 and SGC7901 cells and primordial tumours (Fig. 1H and I). The anticancer drug taxol, which was administered to cancer cells and labelled with FITC, accumulated less in the A2780DX5 and SGC7901R cells (the bright dots in Fig. 1J and L), and the fluorescence was weaker than that in the A2780 and SGC7901 cells (Fig. 1K). These results demonstrate that the A2780DX5 and SGC7901R cells and some recurrent cancers are useful for studying MDR in cancer.

### ABCB1 can be identified as a direct target of miR‐495

**Figure 2:**
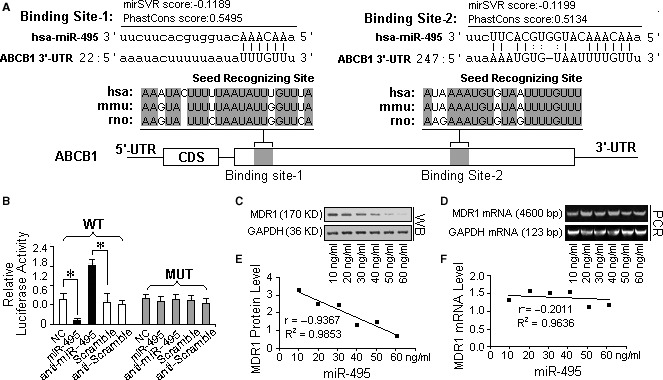
*ABCB1* was identified as a direct target of miR‐495. (**A**) A schematic description of the hypothetical duplexes formed by the interactions between the binding sites in the ABCB1 3′‐UTR and miR‐495. The mirSVR scores (−0.1199, −0.1199) and PhastCons scores (0.5495, 0.5134) of the two hybrids are within the range of genuine miRNA‐target pairs. Two seed recognition sites were found in the 3′‐UTR, and the nucleotides in these regions are highly conserved across humans, mice and rabbits. (**B**) The luciferase reporter activity of the vector containing the mutated miR‐495 binding sites in the ABCB1 3′‐UTR was unaffected by miR‐495. In contrast, the luciferase reporter activity of the plasmid containing the wild‐type MDR1 3′UTR sequence was increased more than 75% in A2780DX5 cells cotransfected with a transfection control plasmid (β‐gal) and anti‐miR‐495, but it was unaffected by the knockdown of miR‐495, compared with the cells treated with the negative control RNA, suggesting a specific binding between miR‐495 and the mRNA of MDR1. (**C**) Dose‐dependent changes in the expression of the MDR1 protein in A2780DX5 cells expressing the miR‐495 mimic. (**D**) Dose‐dependent changes in the expression of the MDR1 mRNA in A2780DX5 cells transfected with the miR‐495 mimic. (**E** and **F**) Pearson's correlation scatter plots of the fold change of the levels of miR‐495 and *ABCB1* protein or mRNA in A2780DX5 cells. There is an inverse correlation between the miR‐495 levels and MDR1 levels, but no significant difference can be observed between the MDR1 mRNA levels of the differently treated cells, implying that miR‐495 inhibited the translation of the MDR1 mRNA but that it did not induce degradation of the mRNA itself. *Student's *t*‐test *(paired, one tailed), P <* 0.05*, n =* 3.

Using the algorithms described above, miR‐214, miR‐129‐5p, miR‐875‐5p, miR‐223, miR‐186, miR‐374b, miR‐374a, miR‐495, miR‐429, miR‐200b, miR‐200c, miR‐155, miR‐873 and miR‐381 were predicted by one or more algorithm to target the mRNA of MDR1 (ABCB1). Among these miRNAs, miR‐495 was found to target two sites in the 3′‐UTR of the ABCB1 mRNA sequence. The mirSVR scores of the hybrids were −0.1189 and −0.1199, and the PhastCons scores were 0.5495 and 0.5134, which are well within the range of genuine miRNA‐target pairs, suggesting that miR‐495 can potentially down‐regulate MDR1 expression 11. Perfect base pairings occurred between the seed region (the core sequence that encompasses the first 2–8 bases of the mature microRNA) and the cognate targets. The miR‐495 binding sequences in the MDR1 3′‐UTR are highly conserved across humans, mice and rabbits (Fig. 2A). Moreover, mutation of the predicted miR‐495 binding sites in the MDR1 3′‐UTR resulted in no change in the activity of the luciferase reporter after overexpression of miR‐495 (Fig. 2B). In contrast, when the wild‐type MDR1 3′UTR‐containing plasmid was transfected into A2780DX5 cells along with a transfection control plasmid (β‐gal) and anti‐miR‐495, the knockdown of miR‐495 resulted in a 75% increase in luciferase reporter activity compared to cells treated with the negative control RNA (Fig. 2B).

MicroRNAs are generally thought to have expression patterns that are opposite to that of their targets 12, 13, 14. Using Pearson's correlation scatter plots, an inverse correlation between the miR‐495 levels and ABCB1 protein (MDR1) levels (*r* = −0.9367 and −0.2011, Fig. 2C and E), but not mRNA levels (Fig. 2D and F) was observed in the MDR cell line A2780DX5. These findings suggest that the two identified binding sites strongly contribute to the miRNA‐mRNA interaction to mediate the post‐transcriptional repression of ABCB1 expression.

### miR‐495 can reduce MDR1 and sensitize cancer cells to the combination of taxol and doxorubicin

**Figure 3:**
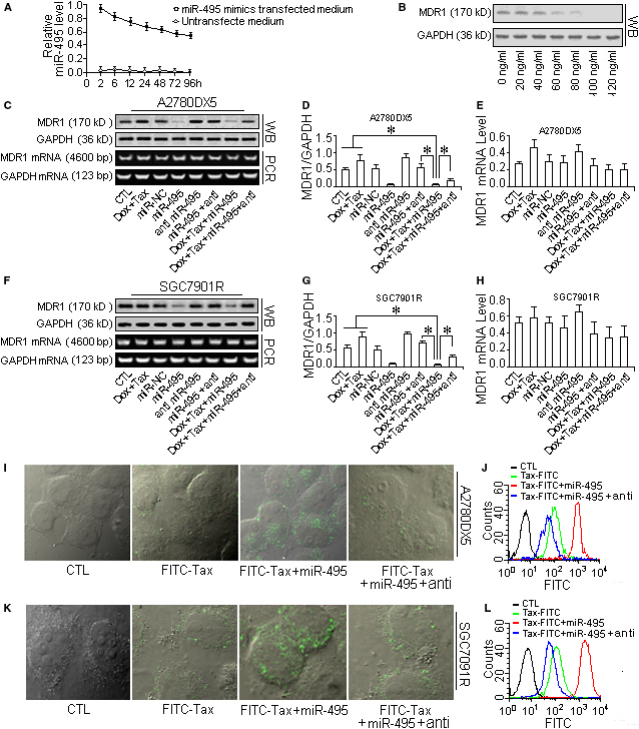
miR‐495 mimic injections down‐regulated MDR1 and reduced the efflux of taxol from the cells. To verify the role of MDR1 in drug efflux, the miR‐495 mimic was overexpressed in MDR cells by transfection, and then, the MDR1 inhibition was verified with by Western blot analysis, and the drug accumulation was measured using the indicator FITC‐labelled taxol. (**A**) Half‐life assay of the miR‐495 mimic in the cell culture medium. (**B**) Dose‐dependent effect of the miR‐495 mimic on the suppression of MDR1 expression in A2780DX5 cells. The MDR1 protein level decreased with the transfection of increased amounts of the miR495 mimic. (**C**) The protein and mRNA levels of MDR1 in differently treated A2780DX5 cells. (**D**) The quantification of the MDR1 protein in cells. (**E**) The quantification of the MDR1 mRNA in cells. (**F**) The protein and mRNA levels of MDR1 in differently treated SGC7901R cells. (**G**) The quantification of the MDR1 protein in differently treated SGC7901R cells. (**H**) The quantification of the MDR1 mRNA in differently treated cells. Transfecting miR‐495 into the two MDR1‐rich cell lines only reduced the MDR1 protein expression, whereas it did not affect the mRNA expression. (**I** and **K**) Using the FITC‐labelled taxol as an indicator to measure the ability of cells to absorb the drug, we demonstrated that the MDR1 reduction caused a decrease in drug efflux, and more FITC‐taxol accumulated in the A2780DX5 and SGC7901R MDR cancer cells. (**J** and **L**) miR‐495 administration strengthened the fluorescence (which was emitted by FITC‐taxol in the cells (the red histogram). The miR‐495 antisense RNA abolished the drug accumulation, and fewer drug molecules were retained in the cells, resulting in weakened fluorescence (blue histogram). *Student's *t*‐test *(paired, one tailed), P <* 0.05*, n =* 5.

**Figure 4:**
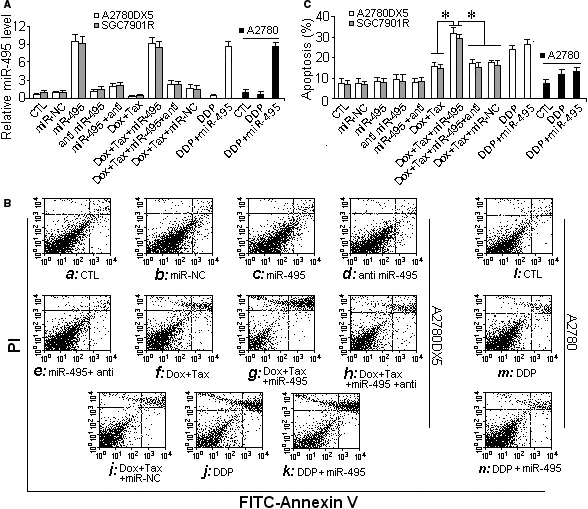
miR‐495 sensitized MDR cells to taxol‐doxorubicin mixtures. To promote the toxic effect of drugs on MDR cancer cells, the MDR1 expression in cells resistant to the taxol‐doxorubicin mixture was suppressed by transfecting the cells with the miR‐495 mimic. (**A**) Relative miR‐495 levels in the cell lines A2780DX5 and SGC7901R and the cisplatin‐resistant cell line A2780C. (**B**) The sensitivity to taxol was assayed by flow cytometry to measure cell apoptosis after the cells were transfected with the miR‐495 mimic. (**C**) The quantification of cell apoptosis. The miR‐495 mimic increased the taxol‐doxorubicin‐induced cell apoptosis but had less of an effect on the cisplatin‐treated cells. *Student's *t*‐test *(paired, one tailed), P* < 0.05*, n* = 5.

Western blotting and mRNA assays both demonstrated that the miR‐495 mimic inhibited the expression of ABCB1 protein but not ABCB1 mRNA (shown in Fig. 2B–F and Fig. 3B–H). As a result, drug efflux was reduced, which allowed more anticancer agents to accumulate in the cells (Fig. 3I, J and K, L). In addition, the effects of the drugs on the MDR cells were strengthened (Fig. 4B: g and Fig. 4C). Moreover, the activity of caspase‐3 also increased, which indicated increased cell apoptosis (Fig. S2A), and more cells were dyed by trypan blue (Fig. S2B and C). In contrast, A2780DX5 MDR cells treated with the drug mixture alone exhibited only a 15% apoptosis rate in 15 hrs (Fig. 4B: f, Fig. 4C and Fig. S2), and the MDR1 levels appeared higher than that of the control cells (Fig. 3C, D and F, G). miR‐495 overexpression alone did not cause obvious additional increases in cell apoptosis (Fig. 4B: c, and Fig. 4C) or loss of cell viability (Fig. S2), although it did decrease the level of MDR1 (Fig. 3B–D and F, G).

The therapeutic effect of the non‐MDR1 substrate drug cisplatin was not reversed by miR‐495 overexpression. As Figure 4B: l–n and Fig. 4C shows, upon administration of the miR‐495 mimic, the apoptosis of the cisplatin‐stressed (cisplatin is not a substrate of MDR1) A2780C cells (an endometrial cancer cell line resistant to cisplatin 15) did not increase or decrease much compared with the cells treated only with cisplatin, and a similar result was observed in cisplatin‐stressed A2780DX5 cells (Fig. 4B: j, k and Fig. 4C), suggesting that miR‐495 is ABCB1 specific.

**Figure 5:**
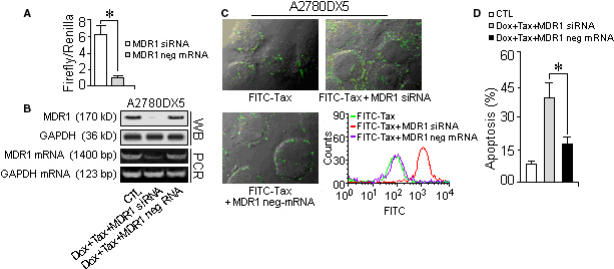
RNAi confirmed that the depletion of MDR1 can decrease drug efflux and improve chemotherapeutic effects. MDR1‐rich MDR cells were transfected with MDR1‐specific siRNA, and then, the MDR1 level was evaluated using Western blot analysis. The drug exclusion ability was indicated by FITC‐labelled taxol. After the depletion of the MDR1 transporter, the drug cytotoxicity was assayed by flow cytometry and indicated by the apoptosis rate. (**A**) Luciferase reporter activity was increased in A2780DX5 cells transfected with MDR1 siRNA. (**B**) MDR1 siRNA transfection resulted in the suppression of MDR1 expression. (**C**) The FITC‐Taxol accumulation increased in the MDR1 siRNA‐transfected cells, indicating that MDR1 inhibition caused a reduction in drug efflux and intensified the drug fluorescence (red curve) compared with that of the no siRNA‐treated cells (green curve). (**D**) RNAi‐induced MDR1 depletion promoted the apoptosis of doxorubicin–taxol‐treated cells. *Student's *t*‐test *(paired, one tailed), P <* 0.05*, n =* 5.

The MDR1 inhibition and accordingly the chemotherapeutic effect could be reversed if the transfected miR‐495 mimic was counteracted by antisense RNA (Fig. 4B: e, i and Fig. S4). Furthermore, siRNA‐mediated ABCB1 silencing (Fig. 5A and B) supported the assumption that MDR1 depletion can attenuate drug loss (Fig. 5C) and that subsequently accelerate cellular apoptosis or decrease the viability of drug‐stressed MDR cells (Fig. 5D and Fig. S3).

**Figure 6:**
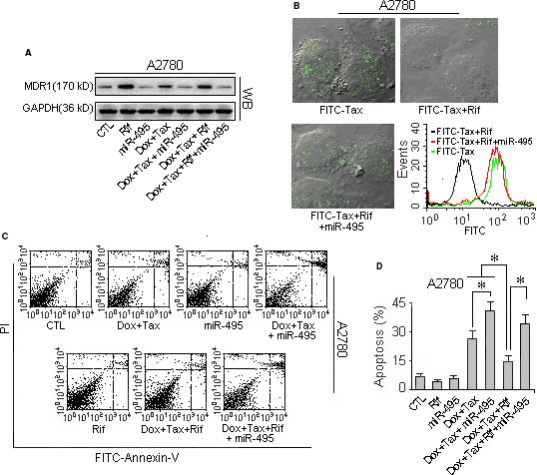
Promoting MDR1 expression with rifampicin rendered the sensitive cell line A2780 drug resistant. To determine the role of MDR1 in the drug efflux in cells, the expression of the transporter MDR1 in the taxol‐doxorubicin‐sensitive cell line A2780 was promoted by rifampicin treatment. The drug efflux ability was verified with the indicator FITC‐Taxol, and the drug resistance enhancement was indicated by the apoptosis rate assessed by flow cytometry. (**A**) The MDR1 expression level in the sensitive cell line A2780 was increased after 2 weeks of rifampicin induction, but the increase could be offset by miR‐495 expression. (**B**) Rifampicin induced a decreased response to FITC‐taxol in the sensitive cell line A2780 and accordingly abated the drug fluorescence (the black curve) in cells compared with the rifampicin‐free cells. (**C**) The flow cytometry of the cells indicates that using rifampicin to promote the MDR1 expression in the sensitive cells A2780 caused the sensitive cells to become resistant to drugs. However, miR‐495 could reverse the rifampicin‐induced MDR, and the induced resistant A2780 cells regained their sensitivity to taxol. (**D**) The quantification of the apoptosis rates measured by flow cytometry. *Student's *t*‐test *(paired, one tailed), P <* 0.05*, n =* 5.

Promoting the expression of MDR1 with rifampicin in A2780 cells (in which the baseline MDR1 expression is low, making these cells sensitive to taxol) strengthened the MDR of these cells. Upon rifampicin administration, the expression of the drug transporter MDR1 in these cells increased many fold (Fig. 6A), and as a result, these sensitive cells became resistant to taxol. As shown in Figure 6B–D and Figure S6, compared with the rifampicin‐free A2780 cells, the FITC‐taxol in the rifampicin‐treated cells decreased, and more cells survived in the taxol‐containing medium. However, when the cells with enhanced drug transporter expression were transfected with the miR‐495 mimic, the level of MDR1 was decreased (Fig. 6A), and the accumulation of FITC‐taxol was reduced to levels similar to those of cells treated with the drug mixture alone (Fig. 6B). In addition, miR‐495 overexpression resulted in increased apoptosis (Fig. 6C and D) and reduced viability (Fig. S4) in rifampicin‐treated cells.

### MiR‐495 can deter the development of MDR tumours *in vivo*

**Figure 7:**
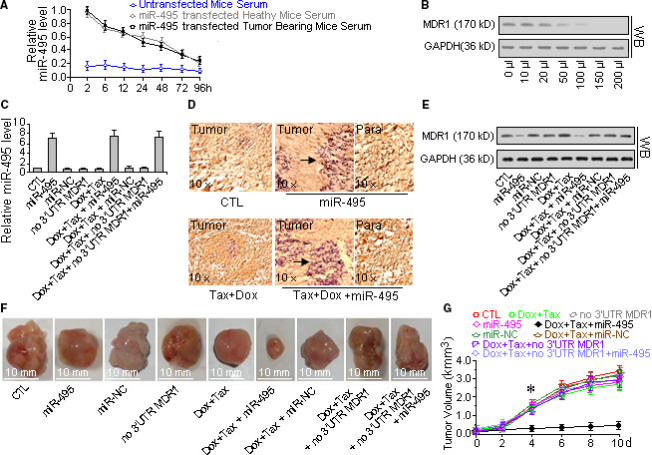
Inhibiting MDR1 with miR‐495 sensitized MDR tumours to the combination of taxol and doxorubicin. To understand how the inhibition of MDR1 by miR‐495 can improve the tumour growth inhibition effect of chemotherapy, a PEI‐conjugated miR‐495 mimic was injected around the tumours, and the expression level of MDR1 and its *in situ* distribution were determined by Western blot and immunohistochemistry analysis, respectively. (**A**) Half‐life assay of the miR‐495 mimic *in vivo*. Two days after the injection of the miR‐495 mimic, half of the original concentration of the miR‐495 mimic remained in the serum, which is nearly four times denser than that of the normal, untreated mice, suggesting that a 2‐day interval of miR‐495 mimic injection is appropriate for the overexpression of miR‐495. (**B**) Dose‐dependent effect of the miR‐495 mimic on the suppression of MDR1 in tumours *in vivo*. The concentration of the miR‐495 mimic was 2 μg/ml, and these results indicate that injections of 150 μl of the PEI‐conjugated miR‐495 mimic every other day are enough to inhibit the expression of MDR1 in tumours. (**C**) miR‐495 levels in the various treated tumours. (**D**) *In situ* hybridization staining of miR‐495 hybridization. The transfected miR‐495 mimic was distributed primarily around the tumours and in the adjacent tissues and was less frequently localized further away from the tumour. (**E**) The MDR1 levels in the treated tumours. The MDR1 expression decreased in tumours treated with the miR‐495 mimic. (**F**) Size comparison of the treated tumours. (**G**) Growth curve of the tumours. Aided by miR‐495, the growth of the tumours slowed under the taxol‐doxorubicin chemotherapy. *Student's *t*‐test *(paired, one tailed), P <* 0.05*, n =* 5.

The *in vivo* experiments were performed with mice‐bearing tumours originating from A2780DX5 MDR cells. Before the formal experiment, half‐life and dose‐dependent assays were performed. Figures 7A and B show that a 2‐day interval between treatments of 100 μl of 2 μg/ml miR‐495 mimic is reasonable. The staining of miR‐495 indicates that the transfected miR‐495 mimic distributes within or around the tumours at the injection sites (Fig. 7D). The taxol‐doxorubicin mixture did not greatly affect tumour growth. In contrast, when the drug mixture was accompanied with the miR‐495 mimic, the curative effect was enhanced significantly. As Figure 7F and G show, in the saline‐injected mice, the tumours grew rapidly from the 3rd to the 6th day and by the 10th day, the tumours reached 3.1 kmm^3^ in volume, nearly 18 times greater than on day 0. The drug mixture alone did not obviously slow the development of the MDR tumours. By the 10th day, the tumours volumes were 16 times larger than on day 0 and were only 15% smaller than the saline‐injected tumours. However, when the chemotherapy drugs were aided by injections of the synthesized miR‐495 mimic, the tumours were nearly dormant, and by the final day, their volumes measured only 0.24 kmm^3^ on average, with a mere 11% increase since the treatment on day 0. Meanwhile, the expression of MDR1 declined significantly (Fig. 7E).

The miR‐495 mimic had little effect on the drug resistance of the tumours when applied with the 3′‐UTR‐lacking MDR1 mRNA plasmids, that is, the injection of 3′‐UTR‐lacking MDR1 mRNA plasmids resulted in higher MDR1 expression in the tumours, which led to increased tumour growth during taxol‐doxorubicin chemotherapy even when the miR‐495 mimic was also injected (Fig. 7F and G).

## Discussion

Drug resistance decreases the effectiveness of agents against cancer. Treatment methods such as chemotherapy have been adopted to combat drug resistance 12, 13, 14, 16. Although achievements have been made, many cancers still develop MDR, which weakens the power of drugs in cancer therapy 15. Even drug combinations cannot address this problem. Worse, the dose used in chemotherapy is often lethal to cells and poisons the body.

Multidrug resistance can have many causes, including increased drug efflux, alterations in drug targets, DNA repair, cell cycle regulation and evasion of apoptosis 17, 18, 19, 20.

MDR1, a well‐known multidrug–efflux transporter, can pump cytotoxic reagents out of the cell 21, 22, and therefore, its overexpression in cancer cells is correlated with the MDR phenotype 21. Moreover, drugs that can activate the MAPK‐ERK/JNK pathways can also initiate the transcription of the ABCB1 gene 23, 24, 25, 26, 27, 28, 29, 30, thus inducing MDR. To circumvent this unfavourable outcome, Perez and David *et al*. used siRNA to deplete the MDR1 mRNA and sensitize ovarian and lung cancer cells to chemotherapy 31, 32, 33. miRNAs play key regulatory roles in the development, differentiation and apoptosis of normal cells, as well as in the determination of the final phenotype of cancer cells, affecting carcinogenesis and metastatic potential 34.

Remarkably, accumulating studies have reported that microRNAs play key roles in the regulation of cancer cells with intrinsic/acquired drug resistance through varied mechanisms. Xu^a^ *et al*. reported that miRNAs are involved in the regulation of chemoresistance to ovarian cancer, colorectal cancer, non‐small cell lung cancer or breast cancer by targeting mTOR/P70S6K1 and MAP/ERK kinase kinase 1 (MEKK1), among others. In addition, using the targeting miRNAs, they resensitized drug‐resistant cells to anticancer agents 35, 36, 37, 38, 39, 40.

**Figure 8:**
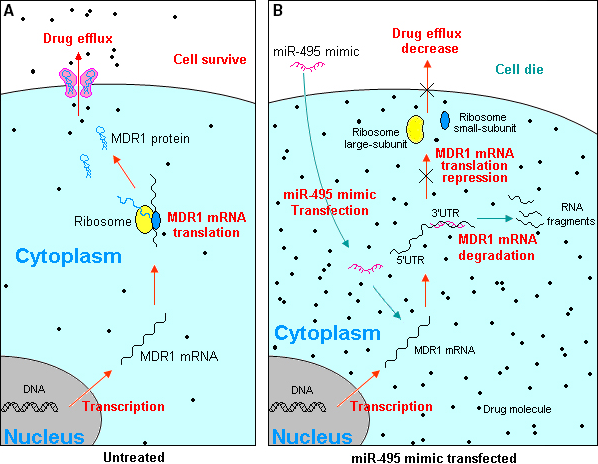
Schematic diagram illustrating miR‐495 decreasing drug efflux *via* inhibiting MDR1. (**A**) Without intransfected miR‐495, the transcribed MDR1 mRNA can be translated to MDR1 protein to assemble drug transporter. It helps the cell pump out the absorbed drugs, which exempts cell from cytotoxicity; (**B**) over intransfected exotic miR‐495 mimics complementary bind to MDR1 mRNA, causes MDR1 mRNA degradation or MDR1 mRNA translation inhibition. As the consequence, the membrane drug transporter become scarce, it decreases the drug efflux, which leading to the cellular drug accumulates denser and with the result that the drug effect on MDR cancer cell improvement.

Here, as the diagram Figure 8 shows, by complementary binding to the 3′‐UTR of the mRNA of MDR1 (Fig. 2) in MDR cancer cells and some recurrent tumours, miR‐495 blocked MDR1 translation, which depressed the expression of MDR1 and consequently decreased drug efflux. The combination of doxorubicin and taxol is often used at a lethal dose, and worse, the mixture often promotes the expression of MDR1 23, 24, 25, 26, 27, 28, 29, 30 (Fig. 3), which expels the drugs from cells and shields cancer cells from cytotoxicity. In contrast, the overexpression or knockdown of miR‐495 is not embryonically fatal 41, although it may cause cellular abnormalities.

The role of miR‐495 in tumour suppression is not exclusively due to MDR1 inhibition. As predicted by the online software TargetScan, more than 10 000 genes may be targeted by miR‐495 (http://www.targetscan.org/cgi-bin/targetscan/vert_61/targetscan.cgi?species=Human%26gid=%26mir_sc=%26mir_c=%26mir_nc=%26mirg=hsa-miR-495), including some involved in cell growth or proliferation. Examples include PTEN (phosphatase and tensin homolog), CDCA7 (cell division cycle associated 7), CCNA2 (cyclin A2), CDK14 (cyclin‐dependent kinase 14), MAP3K2 (mitogen‐activated protein kinase 2) and MAP4K4, along with other genes encoding cell death or differentiation factors, such as PDCD10 (programmed cell death 10) and CFLAR (CASP8 and FADD‐like apoptosis regulator). Therefore, the effect of tumour promotion or inhibition may be the comprehensive result of involved factors. For example, Li, Xu^b,c^ and Ai reported that miR‐495 suppresses cell growth or migration in prostate cancer, endometrial cancer and lung cancer *via* the down‐regulation of FOXC1 expression or by targeting Akt and mTOR signalling 42, 43, 44, 45.

In addition to miR‐495, there are other microRNAs such as miR‐302a/b/c/d, miR‐122 and others that can reverse MDR *via* inhibiting MDR1. Zhao *et al*. sensitized breast cancer cells and HCC cells to adriamycin and vincristine in their studies 46, 47.

Forcing targeting miRNAs to regulate chemoresistance is a novel therapy for the treatment of MDR cancer. Compared to chemotherapy, miR‐495 is cytoendogenous with low toxicity. In addition, now that physiologically active miR‐495 mimics can be mechanically synthesized, the clinical application of miR‐495 is a clear possibility.

## Disclosure statement

The authors have no conflict of interest.

## Supporting information


**Figure S1** Caspase‐3 (DEVD‐pNa) activity and MTT assay.

**Figure S2** Caspase‐3 activity was improved and cell viability decreased under taxol‐doxorubicin mixture stress after the miR‐495 administration.

**Figure S3** Caspase‐3 activity was enhanced and cell viability decreased under taxol‐doxorubicin stress after depleting MDR1 with siRNA.

**Figure S4** The activity of caspase‐3 of sensitive cells A2780 decreased and the viability of taxol‐doxorubicin stressed cell increased after the administration of rifampicin.
