# Supplementary figures and images for: miR‐495 sensitizes MDR cancer cells to the combination of doxorubicin and taxol by inhibiting MDR1 expression
Source: J Cell Mol Med. 2017 Apr 14;21(9):1929–43. doi: 10.1111/jcmm.13114 (PMC5571520; doi:10.1111/jcmm.13114)

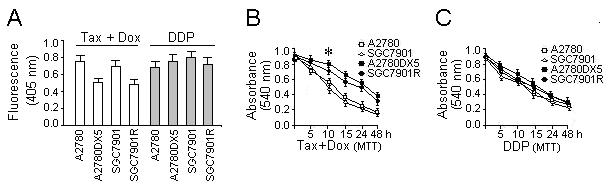

Supplement: Supplementary file 1 — Figure S1 Caspase‐3 (DEVD‐pNa) activity and MTT assay. [file JCMM-21-1929-s001.jpg]

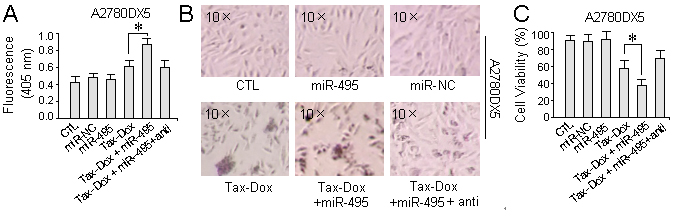

Supplement: Supplementary file 2 — Figure S2 Caspase‐3 activity was improved and cell viability decreased under taxol‐doxorubicin mixture stress after the miR‐495 administration. [file JCMM-21-1929-s002.jpg]

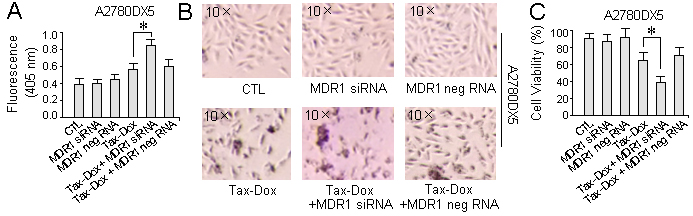

Supplement: Supplementary file 3 — Figure S3 Caspase‐3 activity was enhanced and cell viability decreased under taxol‐doxorubicin stress after depleting MDR1 with siRNA. [file JCMM-21-1929-s003.jpg]

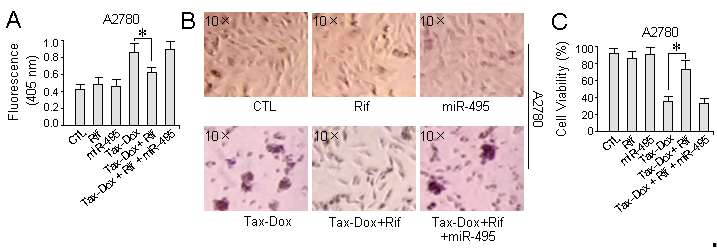

Supplement: Supplementary file 4 — Figure S4 The activity of caspase‐3 of sensitive cells A2780 decreased and the viability of taxol‐doxorubicin stressed cell increased after the administration of rifampicin. [file JCMM-21-1929-s004.jpg]
