# Supplementary material for: miR‐495 sensitizes MDR cancer cells to the combination of doxorubicin and taxol by inhibiting MDR1 expression
Source: J Cell Mol Med. 2017 Apr 14;21(9):1929–43. doi: 10.1111/jcmm.13114 (PMC5571520; doi:10.1111/jcmm.13114)
Supplement: Supplementary file 5 [file JCMM-21-1929-s005.doc]

**Supplement**

**Supplemental figure legends**

**Fig. S1 Caspase-3 (DEVD-pNa) activity and MTT assay**

**A)** Caspase-3 (DEVD-pNa) activity of the cells was assayed by the in vitro fluorogenic caspase. **B)** MTT assay of taxol-doxorubicin-stressed cells. **C)** MTT assay of cisplatin-stressed cells.

**Fig. S2** **Caspase-3 activity was improved and** **cell viability decreased under taxol-doxorubicin mixture stress after the miR-495 administration.**

**A)** Caspase-3 activity determination. **B)** Cell viability was determined by the absence of trypan blue dye. **C)** The quantification of cell viability was determined by the absence of trypan blue stain. miR-495 mimics transfection promoted the efficiency of taxol-doxorubicin-combination on A2780DX5 cell killing.

**Fig. S3 Caspase-3 activity was enhanced** **and cell viability decreased under taxol-doxorubicin stress after depleting MDR1 with siRNA.**

**A)** Caspase-3 activity in differently treated A2780DX5 cells. **B)** Cell viability determined based on the absence of trypan blue dye. **C)** The quantification of cell viability determined by the absence of trypan blue stain.

**Fig. S4 The activity of caspase-3 of sensitive cells A2780 decreased and the viability of taxol-doxorubicin stressed cell increased after the administration of rifampicin.**

**A)** Caspase-3 activity in differently treated A2780 cells. **B)** Cell viability determined based on the absence of trypan blue dye. **C)** The quantification of cell viability determined by the absence of trypan blue stain. The viability of the taxol-doxorubicin stressed cells was promoted after the rifampicin administration**.**
